# Supplementary material for: Research Progress on Micro(nano)plastic-Induced Programmed Cell Death Associated with Disease Risks
Source: Toxics. 2024 Jul 5;12(7):493. doi: 10.3390/toxics12070493 (PMC11281249; doi:10.3390/toxics12070493)
Supplement: Supplementary file 1 [file toxics-12-00493-s001.zip › toxics-3078915-supplementary.pdf]

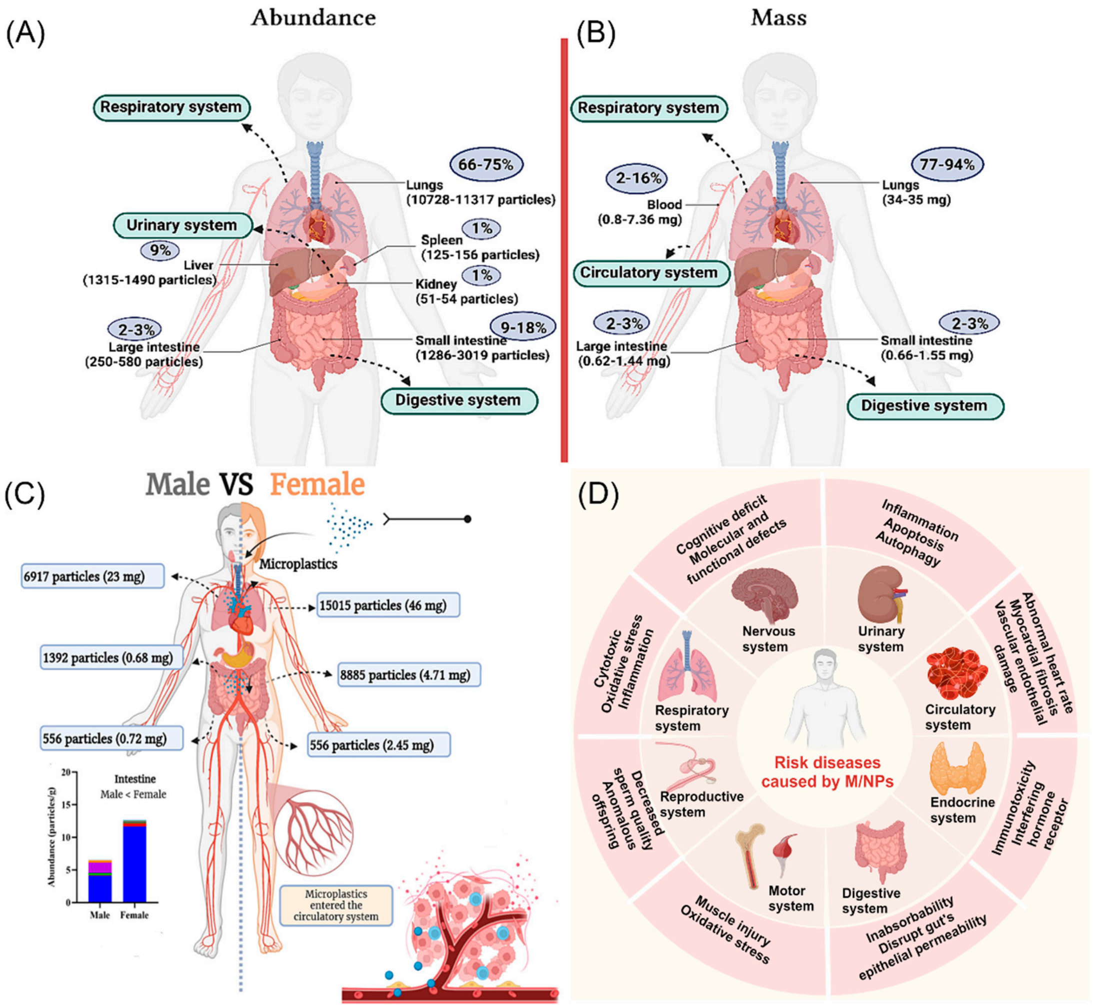

Figure S1. The distribution of MNPs in the human body and potential health risks associated with exposure to MNPs. (A) The total abundance of MNPs in human body. Copyright © 2024 Elsevier. (B) The mass of MNPs in human body. Copyright © 2024 Elsevier. (C) Differences in MNPs accumulation in digestive and respiratory tissues between men and women. Copyright © 2024 Elsevier. (D) The potential health risks may be caused by MNPs. (A), (B) and (C) Adapted with permission from zhu et al. [1]

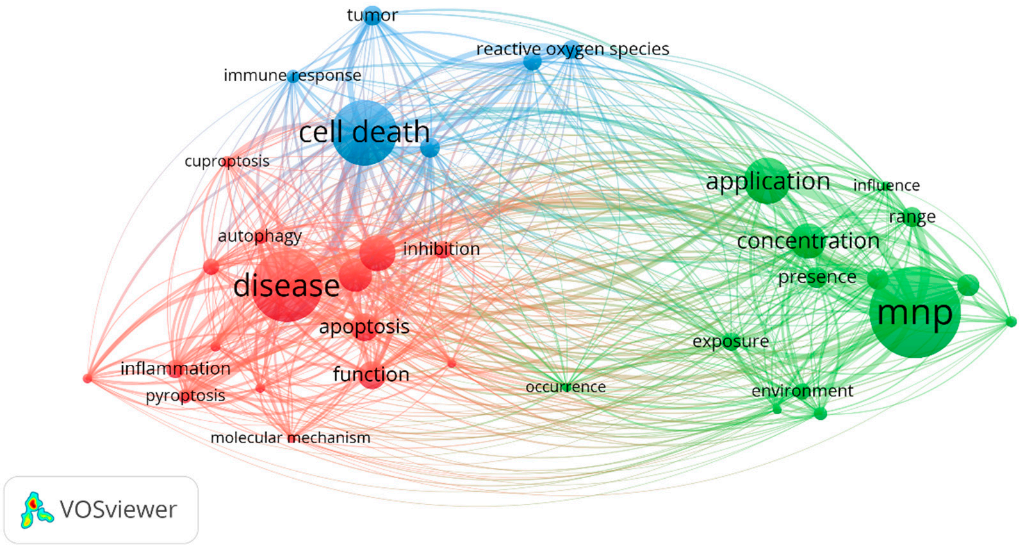

Figure S2. VOSviewer schematic for bibliometric analysis concerning PCD induced by MNPs, and its association with disease. Inclusion and exclusion criteria were applied based on article titles, abstracts, and keywords. Our analysis comprised 241 pertinent articles, employing VOSviewer software to pinpoint significant keywords. Key terms such as "MNP," "cell death," and "disease" have emerged as central themes in current research, underscoring the necessity for further exploration in these domains.

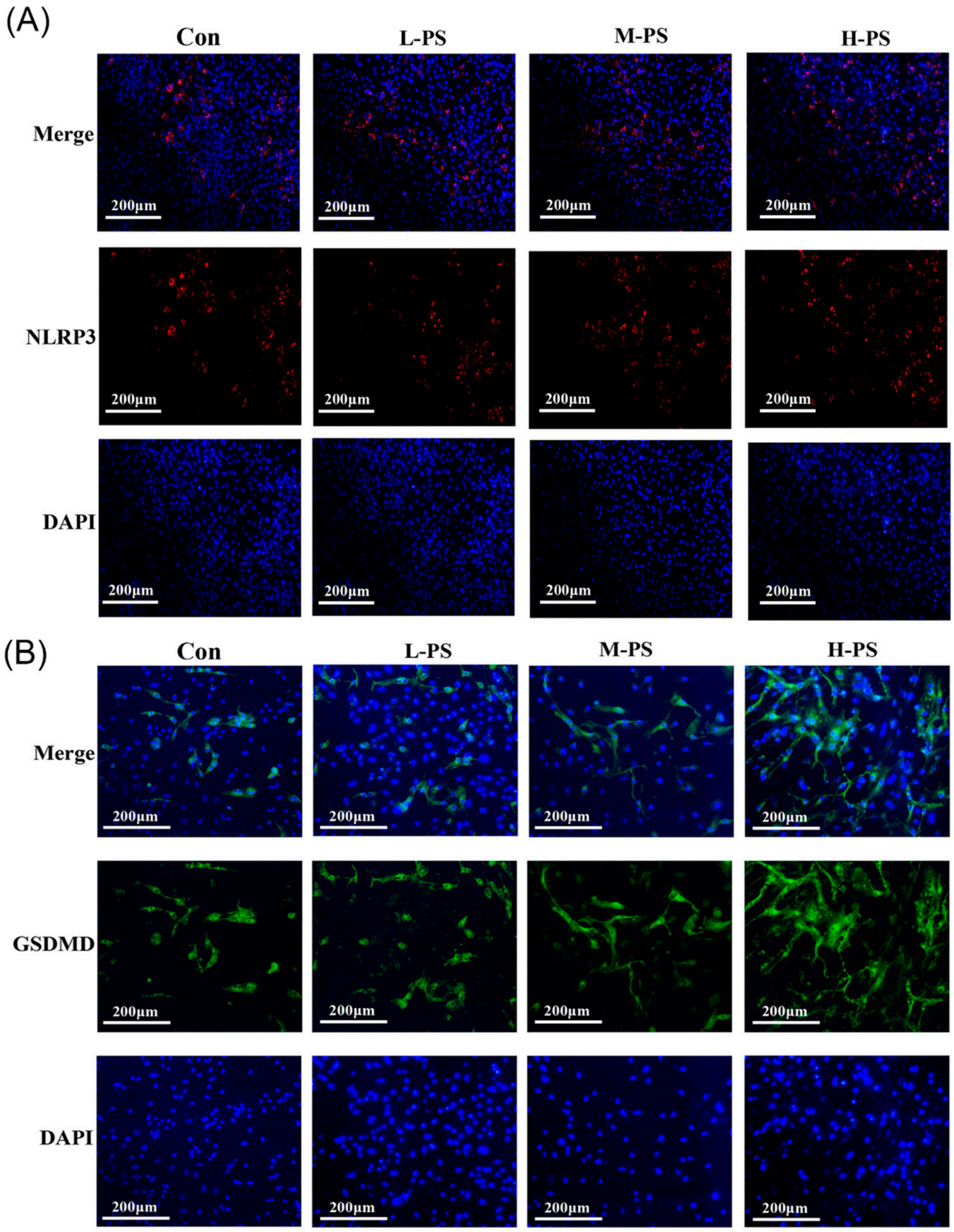

Figure S3. PS-MPs induce pyroptosis in vitro. (A) Immunofluorescence (IF) analysis of NLRP3 expression in primary cardiomyocytes. Copyright © 2022 Elsevier (B) IF analysis of GSDMD expression in primary cardiomyocytes. Copyright © 2022 Elsevier. (A) and (B) Adapted with permission from Zhang et al. [2]

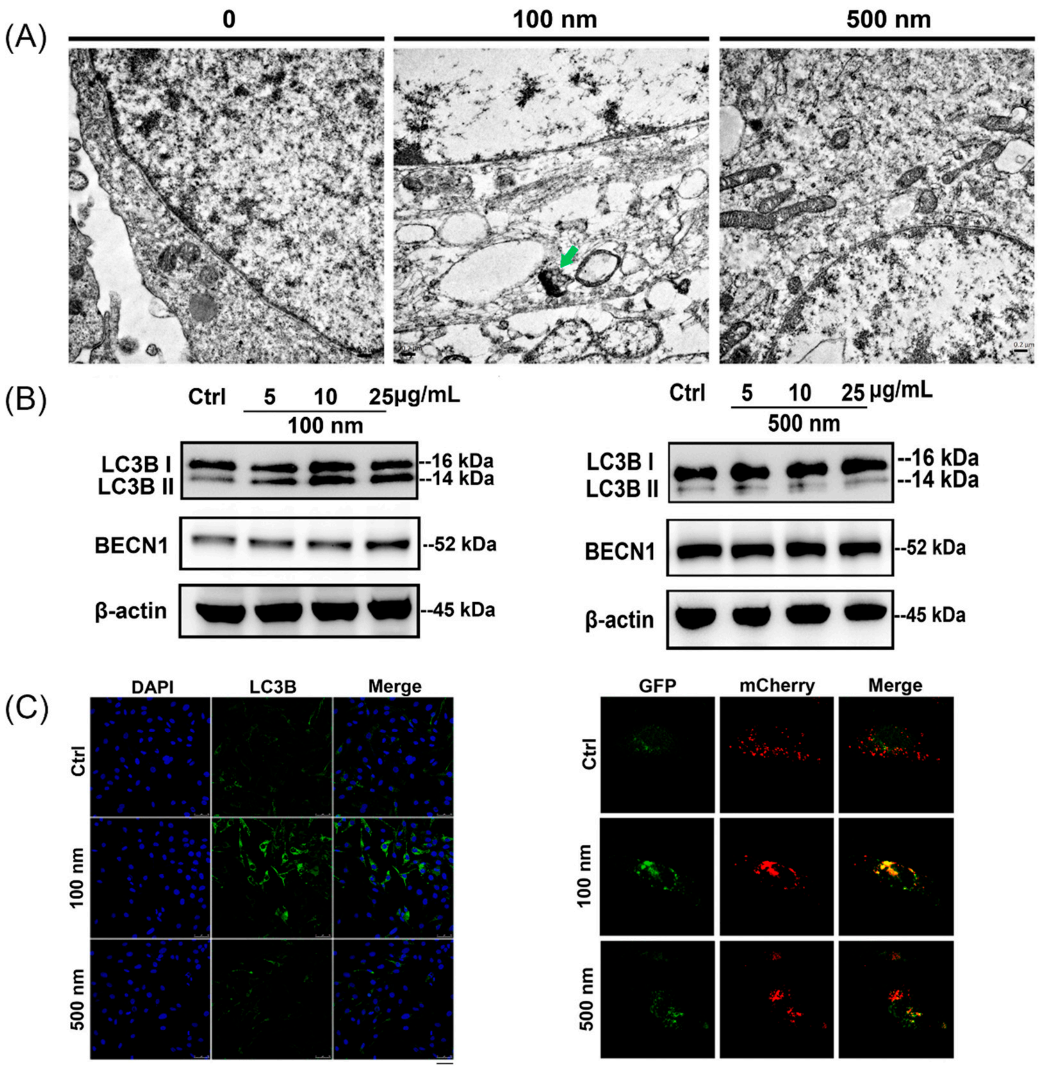

Figure S4. NPs can induce autophagy in cells. (A) Transmission electron microscopy (TEM) revealed the cytoplasmic distribution of 100-nm and 500-nm PS-NPs alongside lysosomes/autophagosomes. Copyright © 2021 Elsevier (B) Western blot analysis was utilized to quantify the protein expression of LC3B and BECN1 following exposure to 100-nm and 500-nm NPs. Copyright © 2021 Elsevier (C) Representative immunofluorescence images showing the intracellular localization of LC3B and mCherry. Copyright © 2021 Elsevier. (A), (B) and (C) Adapted with permission from Lu et al. [3]

Reference

[1] Zhu, L., Kang, Y., Ma, M., Wu, Z., Zhang, L., Hu, R., et al., 2024. Tissue accumulation of microplastics and potential health risks in human. The Science of the Total Environment 915: 170004, Doi: 10.1016/j.scitotenv.2024.170004.

[2] Zhang, Y., Yin, K., Wang, D., Wang, Y., Lu, H., Zhao, H., et al., 2022. Polystyrene microplastics-induced cardiotoxicity in chickens via the ROS-driven NF-κB-NLRP3-GSDMD and AMPK-PGC-1α axes. The Science of the Total Environment 840: 156727, Doi: 10.1016/j.scitotenv.2022.156727.

[3] Lu, Y.-Y., Li, H., Ren, H., Zhang, X., Huang, F., Zhang, D., et al., 2022. Size-dependent effects of polystyrene nanoplastics on autophagy response in human umbilical vein endothelial cells. Journal of Hazardous Materials 421: 126770, Doi: 10.1016/j.jhazmat.2021.126770.
